# Awareness, Knowledge, and Attitude Assessment of Cleft Lip With or Without Palate Management Among Vietnamese Dental and Medical Students: A Cross-Sectional Study

**DOI:** 10.7759/cureus.77197

**Published:** 2025-01-09

**Authors:** Anh Le Kha, Teruyuki Niimi, Hideto Imura, Van Ta Thanh, Son Tong Minh, Ngoc Vo Truong Nhu, Hung Dang Trieu, Tran Thao Phuong, Anar-Erdene Gantugs, Masaaki Ito, Ken Kitagawa, Kayo Hayami, Rie Osakabe, Nagana Natsume, Hiroo Furukawa, Nagato Natsume

**Affiliations:** 1 Division of Research and Treatment for Oral and Maxillofacial Congenital Anomalies, School of Dentistry, Aichi Gakuin University, Nagoya, JPN; 2 School of Dentistry, Hanoi Medical University, Hanoi, VNM; 3 Center for Gene and Protein Research, Hanoi Medical University, Hanoi, VNM; 4 Division of Research and Treatment for Oral and Maxillofacial Congenital Anomalies, School of Dentistry, Aichi Gakuin Dental Hospital, Nagoya, JPN; 5 Center for Cleft Lip and Palate Treatment, Aichi Gakuin University, Nagoya, JPN

**Keywords:** cleft lip with or without palate, dental and medical students, education, questionnaire study, vietnamese

## Abstract

Background

Cleft lip and/or palate (CL/P) are congenital malformations that require multidisciplinary treatment and in-depth knowledge for effective management, especially in countries like Vietnam, where the incidence rate is 1.4 per 1,000 live births. This study aimed to develop and validate a questionnaire to assess the awareness, knowledge, and attitudes of undergraduate medical and dental students at Hanoi Medical University, Vietnam, regarding CL/P management.

Materials and methods

The questionnaire was administered using Google Forms (Google LLC, USA). The study participants were 284 (55.6%) dental students and 227 (44.4%) medical students at Hanoi Medical University, Vietnam. The questionnaire comprised four sections: general information, awareness, knowledge, and attitude assessments.

Results

In the awareness assessment, 97.5% of students were aware of CL/P. However, 84.1% and 66.5% of medical and dental students, respectively, lacked confidence in their current knowledge. Confidence levels increased gradually from third-year to final-year students. Regarding etiology, most students believed genetic factors were the primary cause of CL/P, followed by environmental factors. The most commonly chosen treatment methods were oral, maxillofacial, and plastic surgeries. Dental students showed more interest in CL/P and felt a greater need for additional training in CL/P treatment and management than medical students. Both dental and medical students favored early intervention. However, 19.4% of students were unsure about the optimal time to begin treatment, with this uncertainty being more prevalent among medical students (26%) than among dental students (14.1%) (p<0.001).

Conclusion

This study emphasizes the need for improved education among undergraduate students, especially medical students, to improve CL/P management.

## Introduction

Cleft lip and/or palate (CL/P) is a congenital malformation that affects speech, hearing, and appearance and presents significant long-term health and social integration challenges. As one of the most prevalent craniofacial anomalies, based on a recent meta-analysis, the global prevalence of cleft palate (CP), cleft lip (CL), and CL palate (CLP) in every 1,000 live births was 0.33, 0.3, and 0.45, respectively [1]. CL/P affects approximately one in 500 children in Asia and 1.4 in 1,000 live births in Vietnam [2]. CL and palate are more common in males, while CP is more frequently observed in females [3]. Oral-facial cleft incidence remains over the 30-year period, even with the improvement of diagnosis methods [4].

The etiology of CL/P is considered multifactorial, involving genetic or environmental factors or a combination of both [5]. Environmental factors during pregnancy, such as smoking, alcohol consumption, and exposure to infections, can contribute to CL/P formation. Additionally, folic acid and vitamin deficiencies during pregnancy have been linked to a higher risk of CL/P [6-8]. Several genetic studies have shown an association between genetic factors and oral-facial clefts in the Vietnamese population [9,10].

CL/P imposes significant physical, psychological, and social burdens on affected children, their families, society, and healthcare systems. Children with CL/P have higher rates of psychological issues, such as depression, low self-esteem, and social anxiety, compared to their peers without the condition [11]. Previous studies have shown that adolescents with CL/P experience a lower quality of life and higher levels of stigma compared to healthy adolescents [12,13].

Early detection, referral, and diagnosis significantly improve treatment outcomes and quality of life for children with CL/P and their parents. However, access to CL/P treatment in Vietnam is limited due to a shortage of specialized medical staff and economic difficulties. Consequently, many children with CL/P do not receive timely interventions, leading to suboptimal treatment outcomes and lower treatment quality. Swanson et al. reported that 50% of Vietnamese children with CL/P were unable to access timely surgical treatment [14].

Effective management of CL/P requires a multidisciplinary team approach, including plastic surgery, speech and language therapy, psychology, pediatrics, restorative treatments, and orthodontics [15]. While surgical teams can address most aesthetic issues, such as cleft closure and jaw alignment, postoperative treatments, including speech therapy and psychological support, remain insufficient in Vietnam and other developing countries [15]. Furthermore, research on oral health status in patients with repaired CL/P has revealed high rates of dental caries and gingivitis [16].

Assessing the knowledge of dental and medical students, who represent the next generation of healthcare providers, is crucial for improving their future performance in CL/P management. However, research evaluating the awareness, knowledge, and attitudes of future generations of doctors regarding CL/P management in Vietnam is lacking. This study aimed to develop and validate a questionnaire to assess these factors among undergraduate dental and medical students.

## Materials and methods

Study design and ethical approval

This study was approved by the Ethics Committee of the Japanese Society of Oral Care (E224001). The purpose of the study was explained to all participants, and informed consent was attached to the questionnaire on the first page. Participants have to complete the informed consent before opening the questionnaire section. This study was conducted in accordance with the Checklist for Reporting Results of Internet E-Surveys (CHERRIES) [17] and followed the guidelines of the Declaration of Helsinki.

Sample size calculation

The sample size for this study was calculated using the single proportion formula with a 5% margin of error, 95% confidence level, 2,114 dental and medical students from the third year to the sixth year, and 50% population proportions. The minimum sample was 326.

Participants collection

Questionnaire responses were obtained from all students who were willing to participate in the survey. The participants in this study were collected from April 2024 to June 2024 via Online Google Form (Google LLC, USA), including 227 (44.4%) medical students and 284 (55.6%) dental students, with response rates of 12.7%, and 88.2%, respectively (Table 1).

**Table 1:** Characteristics of participants.

	Medical students					Dental students				
	Third year	Fourth year	Fifth year	Final year	Total	Third year	Fourth year	Fifth year	Final year	Total
Gender										
Male	49 (49%)	13 (52%)	13 (33.3%)	35 (55.6%)	110 (48.5%)	27 (43.5%)	32 (43.8%)	36 (48.6%)	31 (41.3%)	126 (44.4%)
Female	51 (51%)	12 (48%)	26 (66.7%)	28 (44.4%)	117 (51.5%)	35 (56.5%)	41 (56.2%)	38 (51.4%)	44 (58.7%)	158 (55.6%)
Total number of students	100	25	39	63	227	62	73	74	75	284

The inclusion criteria were students from third to sixth grade aged 21 to 24 years old (CL/P knowledge appearing from the third year of the curriculum) at Hanoi Medical University, Hanoi, Vietnam.

Questionnaire

**Figure 1:**
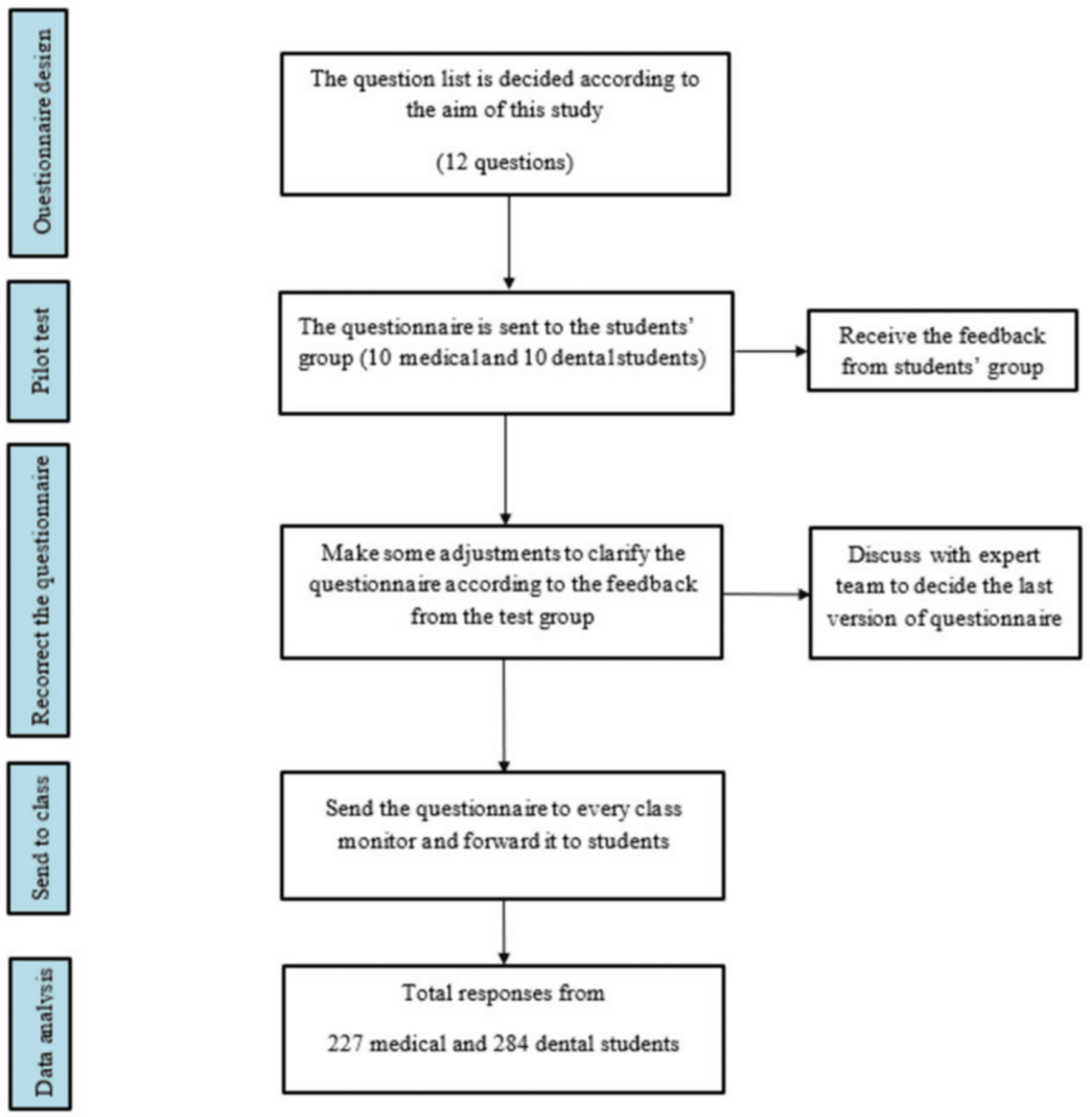
Questionnaire establishment flowchart.

The questionnaire used in our study comprised four sections and 12 closed-ended questions, validated by an expert team on CL/P management from the Division of Research and Treatment for Oral Maxillofacial Congenital Anomalies at Aichi Gakuin University, Japan. The questionnaire was pre-tested by a group of 10 dental and 10 medical students. The expert team adjusted the questionnaire to improve the clarification according to the feedback from this group (Figure 1). The first section was concerned with the details of the respondents and gathered general information, including sex, grade, and major. The second section contained three parts with CL/P-related questions to assess awareness, knowledge, and attitudes regarding CL/P management. 

Data analysis

Descriptive analyses were expressed as number (N) and percentage (%) were analyzed using SPSS 22.0 software (The International Business Machines Corporation (IBM), Armonk, USA). The chi-square test was used to compare the differences between dental and medical students in the response results, with a significance level set at 0.05.

## Results

The study included a total of 511 students, comprising 227 medical students (44.4%) and 284 dental students (55.6%). Among medical students, the largest group was third-year students (100, 44.1%), followed by final-year students (63, 27.8%), fifth-year students (39, 17.2%), and fourth-year students (25, 11%). In dental students, the distribution was relatively balanced across academic years: third-year (62, 21.8%), fourth-year (73, 25.7%), fifth-year (74, 26.1%), and final-year students (75, 26.4%) (Table 1).

Table 2 summarizes the students' responses to questions assessing their awareness of CL/P management. No statistically significant difference was observed between the two groups regarding their awareness of CL/P management, with 97.5% of students being aware of CL/P (96.9% and 97.9% of medical and dental students, respectively). However, confidence in their current knowledge of CL/P management was low, with only 16.9% of medical students and 33.5% of dental students expressing confidence. This difference was statistically significant (P<0.05). Additionally, 71.8% of dental students primarily learned about CL/P through school lectures, compared to 18.1% of medical students (P<0.0001). In contrast, the percentage of medical students who heard about CL/Ps from family members and relatives was higher than that of dental students (33.5% and 20.1%, respectively) (P<0.05). On the other hand, significantly more dental students heard of CL/P doctors and medical staff (64.8% and 54.2%, respectively).

**Table 2:** Students' responses to awareness assessment questions. *The Chi-square test indicates the significant difference at P<0.05.

Questions		Medical students, N=227	Dental students, N=284	Total, N=511	P-value
Before this survey, have you heard about cleft lip with or without palate?					
Yes		220 (96.9%)	278 (97.9%)	498 (97.5%)	0.4884
No		7 (3.1%)	6 (2.1%)	13 (2.5%)	
Are you confident with your current knowledge about cleft lip with or without palate?					
Yes		36 (15.9%)	95 (33.5%)	131 (25.6%)	<0.0001*
No		191 (84.1%)	189 (66.5%)	380 (74.4%)	
Which way did you get information about cleft lip with or without palate? (Multiple choices are available)					
Books and publications	152 (67%)		200 (70.4%)	352 (68.9%)	0.4008
TV	143 (63%)		155 (54.6%)	298 (58.3%)	0.0551
Online social media	152 (67%)		184 (64.8%)	336 (65.8%)	0.6074
Lessons from schools	41 (18.1%)		204 (71.8%)	245 (47.9%)	<0.0001*
Hear from your family members and relatives	76 (33.5%)		57 (20.1%)	133 (26%)	0.0006*
Hear from your friends	76 (33.5%)		77 (27.1%)	153 (29.9%)	0.1184
Hear from doctors and medical staff	123 (54.2%)		184 (64.8%)	307 (60.1%)	0.015*
Other	16 (7%)		8 (2.8%)	24 (4.7%)	0.0247*
Do not know about this disease	7 (3.1%)		6 (2.1%)	13 (2.5%)	0.4884

Table 3 shows the students' responses to the potential etiological factors for CL/P. Genetic factors were identified as the primary cause by 89% of the students (85.5% of the medical students and 91.9% of the dental students). This difference was statistically significant (P<0.05). Additionally, 68.3% of dental students believed that maternal nutritional disorders contributed to CL/P, compared to 51.1% of medical students (P<0.05).

**Table 3:** Students' responses about etiology and typical treatment methods for CL/P. * The Chi-square test indicates the significant difference at P<0.05. CL/P: Cleft lip and/or palate

Questions	Medical students, N=227	Dental students, N=284	Total, N=511	P-value
In your opinion, what are the possible causes of cleft lip with or without palate? (Multiple choices are available)				
Oral care	10 (4.4%)	5 (1.8%)	15 (2.9%)	0.0784
Genetic factors	194 (85.5%)	261 (91.9%)	455 (89%)	0.0206*
Maternal alcohol exposure	125 (55.1%)	132 (46.5%)	257 (50.3%)	0.0537
Maternal smoking exposure	154 (67.8%)	173 (60.9%)	327 (64%)	0.1051
Maternal drug exposure	169 (74.4%)	212 (74.3%)	381 (74.4%)	0.9643
Maternal nutritional disorder	116 (51.1%)	194 (68.3%)	310 (60.7%)	0.0001*
Infectious agents exposure	115 (50.1%)	142 (50%)	257 (50.3%)	0.8821
Geographical and Socioeconomic factors	31 (13.7%)	30 (10.6%)	61 (11.9%)	0.2840
Other	13 (5.7%)	15 (5.3%)	28 (5.5%)	0.8284
What typical treatment methods for cleft lip with or without palate do you know? (Multiple choices are available)				
Plastic surgery	162 (71.4%)	227 (79.9%)	389 (76.1%)	0.024*
Oral and maxillofacial surgery	196 (85.9%)	268 (94.4%)	464 (90.8%)	0.0011*
Orthodontic treatment	59 (25.6%)	131 (46.1%)	190 (37.2%)	<0.0001*
Speech therapy	83 (36.6%)	158 (55.6%)	241 (47.2%)	<0.0001*
Psychological support	81 (35.7%)	142 (50%)	223 (43.6%)	0.0012*
General dental treatment	49 (21.6%)	115 (40.5%)	164 (32.1%)	<0.0001*
Other	5 (2.2%)	9 (3.2%)	14 (2.7%)	0.5062

**Figure 2:**
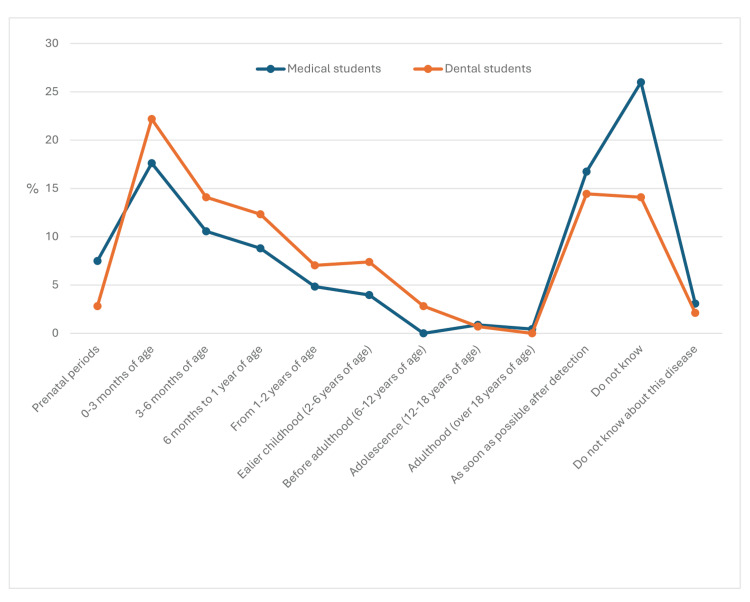
Students’ responses to the question: “When can cleft lip with or without palate patients be started care/treatment?”

Among the students, 72% believed that CL/P could be diagnosed during pregnancy, whereas 17.6% were unsure. Most students preferred that care and treatment begin either immediately upon diagnosis (15.5%) or within the first three months of life (20.2%). However, 19.4% were uncertain about the appropriate time to start treatment, with significantly more medical students (26%) expressing uncertainty than dental students (14.1%) (P<0.001) (Figure 2).

Table 3 also outlines students' preferences for typical treatment methods. Most dental students favored oral and maxillofacial surgery (94.4%), plastic surgery (79.9%), and speech therapy (55.6%). Medical students showed similar preferences at 85.9%, 71.4%, and 36.6%, respectively. Significant differences were found between dental and medical students for all treatment methods (P<0.05).

Most students identified feeding difficulties (90.2%), speech problems (89.4%), and aesthetic issues (90%) as the key challenges faced by individuals with CL/P. Significant differences were observed between dental and medical students in recognizing these difficulties, except for social prejudice (Table 4).

**Table 4:** Students' responses about common difficulties with CL/P patients. * The Chi-square test indicates the significant difference at P<0.05. CL/P: Cleft lip and/or palate

Difficulties	Medical students, N=227	Dental students, N=284	Total, N=511	P-value
Feeding difficulties	197 (86.8%)	264 (93%)	461 (90.2%)	0.0196*
Speech difficulties	193 (85%)	264 (93%)	457 (89.4%)	0.0037*
Dental problems	162 (71.4%)	224 (78.9%)	386 (75.5%)	0.0498*
Esthetic issues	197 (86.8%)	263 (92.6%)	460 (90%)	0.0291*
Hearing loss	23 (10.1%)	70 (24.6%)	93 (18.2%)	<0.0001*
Psychological issues	164 (72.7%)	233 (82%)	397 (77.9%)	0.0113*
Learning disabilities	40 (17.6%)	80 (28.2%)	120 (23.5%)	0.0052*
Social prejudices	151 (66.5%)	200 (70.4%)	351 (68.7%)	0.3447
No difficulty	0 (0%)	0 (0%)	0 (0%)	-
Other	3 (1.3%)	5 (1.8%)	8 (1.6%)	0.6919
Do not know	10 (4.4%)	2 (0.7%)	12 (2.3%)	0.006*
Do not know about this disease	7 (3.1%)	6 (2.1%)	13 (2.5%)	0.4884

Table 5 indicates that 28.5% of dental students examined patients with CL/P during their studies, compared to only 12.8% of medical students (P<0.05). Furthermore, 81.9% of medical students and 91.2% of dental students believed that they needed more training in managing patients with CLPs (P<0.05). However, only 59.5% of medical students and 73.9% of dental students expressed interest in this field (P<0.001). Both student groups preferred school lectures if they had the opportunity to learn more about CL/P management, with 67.4% of medical students and 71.5% of dental students indicating this preference.

**Table 5:** Students' responses according to attitude assessment about cleft lip with or without palate management. * The Chi-square test indicates the significant difference at P<0.05.

Questions	Medical students, N=227	Dental students, N=284	Total, N=511	P-value
Did you examine cleft lip with or without palate patients in the students' period?				
Yes	29 (12.8%)	81 (28.5%)	120 (23.5%)	<0.0001*
Not yet	198 (87.2%)	203 (71.5%)	401 (76.5%)	
Do you need to be trained in cleft lip with or without palate treatment and management?				
Yes	186 (81.9%)	259 (91.2%)	445 (87.1%)	0.0019*
No	41 (18.1%)	25 (8.8%)	66 (12.9%)	
Are you interested in cleft lip with or without palate?				
Yes	135 (59.5%)	210 (73.9%)	345 (67.5%)	0.0005*
No	92 (40.5%)	74 (26.1%)	166 (32.5%)	
If you can get more information about cleft lip with or without palate, which way would you prefer? (Multiple choices are available)				
Lectures	153 (67.4%)	203 (71.5%)	356 (69.7%)	0.3193
Seminars	95 (41.9%)	165 (58.1%)	260 (50.9%)	0.0003*
Social media	109 (48%)	123 (43.3%)	232 (45.4%)	0.2882
Books and publications	116 (51.1%)	112 (39.4%)	228 (44.6%)	0.0084*
Other	7 (3.1%)	8 (2.8%)	15 (2.9%)	0.4884

## Discussion

With the high incidence of CL/P in Vietnam (1.4 per 1,000 live births) [2], there is a critical need for improved multidisciplinary coordination within the healthcare system. However, Vietnam's healthcare system faces significant challenges due to a lack of human resources and comprehensive processes for diagnosing, treating, and managing CL/P [14]. Despite insurance covering up to 70% of surgery costs, over 80% of patients still rely on charity surgeries from domestic or foreign organizations, such as Operation Smile and the Japanese Cleft Palate Foundation [18]. These financial and human resource barriers delay the timing of the first cheiloplasty and palatoplasty, resulting in an average delay until the child reaches three years old [14]. This study aimed to evaluate and improve CL/P management by assessing the awareness, knowledge, and attitudes of dental and medical students who may become part of the CL/P treatment team in the future.

In this study, most (approximately equal proportions) medical and dental students demonstrated a basic awareness of CL/P. Despite CL/P content being included in the curriculum from the third year onward, only 71.8% of dental students and 18.1% of medical students reported learning about it through school lectures (Table 2). This finding suggests that current educational methods are ineffective in transmitting CL/P knowledge to students, especially medical students, highlighting the need to improve educational methods to ensure the broader dissemination of essential information.

While most students were aware of CL/Ps, only 25.6% expressed confidence in their current knowledge of CL/P management, with dental students being significantly more confident than medical students. Given that CL/P predominantly affects dentistry, dental students have greater opportunities to interact with its treatment and management.

Most students identified genetic factors as the primary cause of CL/P, followed by environmental factors, such as drug use, smoking, alcohol consumption, and infections. Previous studies have shown that the etiology of CL/P involves genetic predisposition, environmental factors, or a combination of both, though the precise causes remain unclear [19]. Understanding these causative factors is crucial for improving diagnosis and prevention. Notably, reducing environmental risk factors, including smoking, alcohol use, and poor nutrition, can significantly lower the risk of CL/P in newborns [20].

Early diagnosis is crucial for effective treatment planning, optimizing outcomes in children, and reducing the burden on families and society. Although most students believed that CL/P could be detected during pregnancy, over 20% were unaware of this. Sander et al. conducted a study using ultrasound to detect orofacial clefts. Their findings indicated a high success rate in detecting CL/P, with the highest accuracy observed in identifying bilateral CLP cases [21]. However, isolated CP only (CPO) can be difficult to detect due to ultrasound's limitations in visual assessment. Additionally, MRI can supplement ultrasound by enhancing diagnostic precision, particularly for CPO cases [22,23].

More than 60% of students believed that patients with CL/P should start receiving care and treatment before one year of age, while 26% of medical students and 14.1% of dental students were unaware of the appropriate time to initiate treatment. The diagnosis of CL/P during pregnancy and prenatal counseling plays a significant role in parents' psychological preparation and intervention solutions for children after birth, significantly helping to improve treatment effectiveness [24]. A similar study conducted on Indian dental students revealed that while most students were familiar with CL/P, they lacked specific knowledge, such as treatment time and treatment methods [25].

Oral maxillofacial and plastic surgeries were the two most commonly selected treatment methods among medical and dental students. However, in Vietnam and other developing countries, there is a shortage of specialists in speech and psychology disorders [15], making these treatments less common despite their mention in the curriculum.

Understanding the challenges faced by patients with CL/P is crucial during the examination and assessment process, as well as considering a specific treatment plan for each difficulty. Approximately 90% of the students identified feeding, speaking, and aesthetic challenges as the most common difficulties experienced by CL/P patients. Only 12 students (2.3%) were unaware of the difficulties encountered by the patients with CL/P. A previous study indicated that 67% of children with CPO were at a high risk of feeding difficulties, and it was improved in 79% of CPO children after palatoplasty [26].

Furthermore, dental students showed a stronger interest in CL/P and expressed a greater need for additional training in CL/P treatment and management than medical students. Given this opportunity, students preferred to learn about CL/P through lectures rather than through other methods such as social media or seminars. Therefore, more lectures and the knowledge contained in lectures are required to capitalize on students' enthusiasm in learning through lectures in order to develop students' fundamental knowledge and improve their competency in CL/P management.

This study only focuses on the students at Hanoi Medical University with a sample size that is not too large. We propose further studies at different universities in Vietnam as well as in developed countries with larger sample sizes. This study is the foundation for assessing the current state of education.

## Conclusions

In conclusion, most medical and dental students (approximately equal proportions) were aware of CL/P in this study. However, dental students demonstrated a deeper understanding of the subject, reflecting their more substantial knowledge. They also exhibited greater interest, enthusiasm, and a stronger desire to learn compared to medical students. The study findings emphasize the need for improved CL/P treatment and management education for undergraduate students, especially medical students.

## Appendices

Questionnaire contents

*(A) Informed Consent Form* Study Title: Awareness, Knowledge, and Attitude Assessment on Cleft Lip with or without Palate Management among Vietnamese Dental and Medical Students - A Cross-Sectional Study

Purpose of the study: You are invited to participate in a research study to assess the awareness, knowledge, and attitudes of medical and dental students regarding the management of cleft lip and/or palate (CL/P). The results of this study aim to provide insights that may enhance educational approaches in this field.

Participation details: Participation in this study is voluntary.

You will be asked to complete a questionnaire that will take approximately 10 minutes to complete.

Your responses will be kept confidential and used only for research purposes.

Eligibility: You are eligible to participate if you are a third- to sixth-year student at Hanoi Medical University, aged 21 to 24 years.

Confidentiality: Your responses will remain anonymous and will be stored securely. Only the research team will have access to the data. No identifying information will be linked to the results or published findings.

Risks and benefits: There are no anticipated risks associated with participating in this study.

Your participation will contribute to improving education and awareness regarding CL/P management.

Your participation is entirely voluntary. You may choose to withdraw at any time without penalty or loss of benefits.

Contact Information: If you have any questions or concerns about this study, please contact: LE KHA ANH 1. Division of Research and Treatment for Oral Maxillofacial Congenital Anomalies Aichi Gakuin University, 2-11 Suemori Dori, Chikusa, Nagoya, Japan.

2. School of Dentistry- Hanoi Medical University, Hanoi, Vietnam Lekhaanh268@gmail.com

Consent statement: You have read and understood the purpose and details of the study.

You have had the opportunity to ask questions and receive satisfactory answers.

You agree to voluntarily participate in this study.

Please chose the “Agree” button if you agree to participate in the study after carefully reading all the sections above.

(B) Questionnaire

Part 1: General information 

1.     Name:

2.     Class: 

3.     Age:

4.     Specialization:

a. Dental students

b. Medical students

Part 2: To assess awareness

1. Before this survey, Have you heard about cleft lip with or without palate?

a. Yes

b. No

2. Which way did you get information about cleft lip with or without palate? (Multiple choices are available)

a. Books and publications

b. TV

c. Online social media: Facebook, Instagram, etc

d. Lessons from schools

e. Heard from your family members and relatives

f. Heard from your friends

g. Heard from doctors and medical staff

h. Other

i. Do not know about this disease

3. Are you confident with your current knowledge about cleft lip with or without palate?

a. Yes

b. No

c. Do not know about this disease

Part 3: To assess knowledge

4. In your opinion, what are the possible causes of cleft lip with or without palate? (Multiple choices are available)

a. Oral care

b. Genetic factors

c. Maternal alcohol exposure

d. Maternal smoking exposure

e. Maternal drug exposure

f. Maternal nutritional disorder

g. Infectious agents exposure

h. Geographical and Socioeconomic factors

i. Other

k. Do not know

j. Do not know about this disease

5. Can cleft lip with or without palate be detected prenatally?

a. Yes

b. No

c. Do not know

d. Do not know about this disease

6. When can cleft lip with or without palate patients be started care/treatment?

a. Prenatal periods

b. 0- 3 months of age

c. 3-6 months of age

d. 6 months to 1 year of age

e. From 1-2 years of age

f. Earlier childhood (2-6 years)

g. Before adulthood (6-12 years)

h. Adolescence (12-18 years)

i. Adulthood (over 18 years)

k. As soon as possible after detection

j. Do not know

m. Do not know about this disease

7. What typical treatment methods for cleft lip with or without palate do you know? (Multiple choices are available)

a. Plastic surgery

b. Oral and Maxillofacial surgery

c. Orthodontic treatment

d. Speech therapy

e. Psychological support

f. General dental treatment

g. Other

h. Do not know

i. Do not know about this disease

8. What do you think about the common potential difficulties of cleft lip with or without palate patients? (Multiple choices are available)

a. Feeding difficulties

b. Speech difficulties

c. Dental problems

d. Esthetic issues

e. Hearing loss

f. Psychological issues

g. Learning disabilities

h. Social prejudices

i. No difficulty

k. Other

j. Do not know

m. Do not know about this disease

Part 4: To assess attitude

9. Did you examine cleft lip with or without palate patients in the students' period?

a. Yes

b. Not yet

10. Do you need to be trained in cleft lip with or without palate treatment and management?

a. Yes

b. No

11. Are you interested in cleft lip with or without palate?

a. Yes

b. No

12. If you can get more information about cleft lip with or without palate, which way would you prefer? (Multiple choices are available)

a. Lectures

b. Seminars

c. Social media

d. Books and publications

e. Other
